# Associations of combined lifestyle index with migraine prevalence and headache frequency: a cross-sectional study from the MECH-HK study

**DOI:** 10.1186/s10194-024-01729-y

**Published:** 2024-02-20

**Authors:** Yunyang Deng, Harry Haoxiang Wang, Fei Wan Ngai, Dexing Zhang, Jing Qin, Xiangyan Chen, Yao Jie Xie

**Affiliations:** 1 School of Nursing, The Hong Kong Polytechnic University, Hong Kong SAR, China; 2 School of Public Health, Sun Yat-Sen University, Guangzhou, China; 3 College of Medicine and Veterinary Medicine, The University of Edinburgh, Edinburgh, UK; 4 The Jockey Club School of Public Health and Primary Care, Faculty of Medicine, The Chinese University of Hong Kong, Hong Kong SAR, China; 5 Department of Health Technology and Informatics, The Hong Kong Polytechnic University, Hong Kong SAR, China; 6 Research Centre for Chinese Medicine Innovation, The Hong Kong Polytechnic University, Hong Kong SAR, China

**Keywords:** Lifestyle, Lifestyle index, Lifestyle score, Combined lifestyle, Migraine

## Abstract

**Background:**

Prior research has shown that individual lifestyles were associated with migraine. Yet, few studies focused on combined lifestyles, particularly in Chinese populations. This cross-sectional study aimed to investigate the relationships of a combined lifestyle index with migraine in Hong Kong Chinese women.

**Methods:**

Baseline data from a cohort study named Migraine Exposures and Cardiovascular Health in Hong Kong Chinese Women (MECH-HK) were used for analysis. In total 3510 women aged 55.2 ± 9.1 years were included. The combined lifestyle index comprised eight lifestyle factors: smoking, physical activity, sleep, stress, fatigue, diet, body mass index, and alcohol. Each component was attributed a point of 0 (unhealthy) or 1 (healthy). The overall index was the sum of these points, ranging from 0 (the least healthy) to 8 points (the healthiest). Migraine was diagnosed by the International Classification of Headache Disorders 3rd edition. Additionally, for women with migraine, the data on migraine attack frequency (attacks/month) was collected.

**Results:**

A total of 357 women with migraine (10.2%) were identified. The prevalence of migraine for the 0–3-point, 4-point, 5-point, 6-point, and 7–8-point groups were 18.0% (162/899), 10.9% (86/788), 6.6% (51/776), 6.0% (38/636), and 4.9% (20/411), respectively. In the most-adjusted model, compared to the 0–3-point group, the odds ratios and 95% confidence intervals for the 4-point, 5-point, 6-point, and 7–8-point groups were 0.57 (0.43–0.75), 0.33 (0.24–0.46), 0.30 (0.21–0.44), and 0.25 (0.15–0.41), respectively (all *p* < 0.001). For each component, migraine was significantly associated with sleep, stress, fatigue, and diet; but was unrelated to smoking, physical activity, body mass index, and alcohol. Among women with migraine, per point increase in the combined lifestyle index was associated with a reduced migraine attack frequency (β = − 0.55; 95% confidence interval = − 0.82, − 0.28; p < 0.001).

**Conclusions:**

A combined lifestyle index was inversely associated with migraine and migraine attack frequency in Hong Kong Chinese women. Adhering to a healthy lifestyle pattern might be beneficial to the prevention of migraine attacks. Conversely, it is also plausible that women with migraine might have a less healthy lifestyle pattern compared to those without headaches.

**Supplementary Information:**

The online version contains supplementary material available at 10.1186/s10194-024-01729-y.

## Background

Migraine is a primary headache disorder characterized by head pain, nausea, vomiting, sensory hypersensitivity, or a combination thereof [1]. According to the 2016 Global Burden of Disease study, the global age-standardized prevalence of migraine was 14.4% in all subjects, 18.9% in women, and 9.8% in men [2]. Meanwhile, migraine contributed to 45.1 million years of life lived with disability globally in 2016 [2], which positioned migraine as the second most disabling condition worldwide [3].

Earlier studies have provided robust evidence for the correlations between individual lifestyle factors and migraine. A Norway cohort study identified smoking and physical inactivity as two risk factors for migraine [4]. A meta-analysis showed that individuals with migraine exhibited poorer sleep quality than controls [5]. Another meta-analysis demonstrated that mindfulness-based stress reduction could reduce migraine pain intensity [6]. A meta-analysis also showed that underweight individuals and obese women had an escalated migraine risk than those with normal weight [7]. Furthermore, a cohort study conducted in the United States (US) indicated that tiredness/fatigue could increase migraine risk [8]. Meanwhile, various dietary habits [9–11] and patterns [12, 13] related to migraine were identified.

However, recently, many studies have investigated the relationships of combined lifestyle factors, usually quantified as lifestyle scores/indices, with human health [14–18]. These scores/indices considered potential interactions among lifestyles, providing a holistic basis for policymakers to devise health policies [14–16]. To our knowledge, only two related investigations were performed for migraine, yielding conflicting findings. Specifically, a cross-sectional study in Norway found an elevated prevalence of migraine among students with fewer healthy lifestyles (physical activity, non-smoking, and normal weight) [19]. In contrast, a cross-sectional study in Germany revealed that a 4-item health index (smoking, physical activity, alcohol, and body mass index (BMI)) showed no association with migraine [20].

Nevertheless, lifestyle habits and migraine characteristics differed between Chinese and European populations. For instance, in 2021, the adult daily smoking prevalence rates in China, Norway, and Germany were 21%, 10%, and 17%, respectively [21]. In 2016, the standardized prevalence rates of insufficient physical activity were 14.1% in China and 23.4% in Central and Eastern Europe [22]. Additionally, Chinese and European people exhibited different dietary habits, named the Eastern and Western patterns [23]. The former was characterized by high intakes of whole grains, legumes, fruits, vegetables, and fish, while the latter involved high intakes of refined grains, red and processed meat, butter, high-fat dairy products, and eggs [23]. Furthermore, alcohol consumption per capita in 2015 was higher in Central (11.64), Eastern (11.55), and Western Europe (11.13) compared to East Asia (7.14) [24]. The World Health Organization also employed different BMI cutoffs for European and Asia-Pacific regions [25]. Moreover, from the studies included in a meta-analysis, we observed diverse prevalence rates of sleep disorders during menopause in different regions [26]. Last but not least, in 2016, the age-standardized prevalence of migraine was significantly higher in European regions (15,000–21,000 per 100,000 persons) compared to China (8000-9000) [2]. Consequently, the findings of the two European studies might not be directly applicable to Chinese individuals. Whereas no relevant investigation was conducted within Chinese populations.

Hence, this cross-sectional study aimed to assess the correlation between an 8-item combined lifestyle index (CLI) and migraine among Hong Kong Chinese women.

## Methods

### Study population

**Fig. 1:**
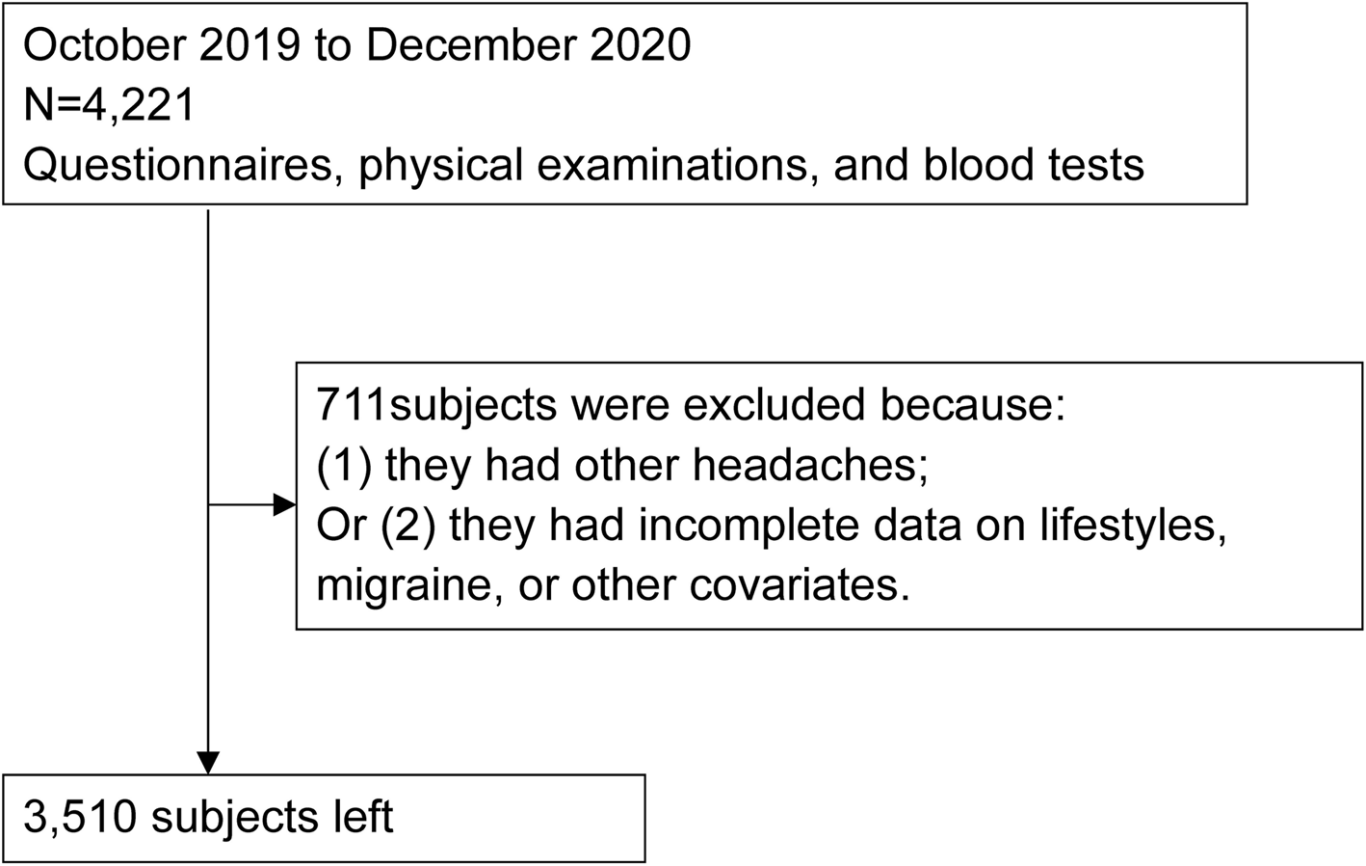
The flow chart for the associations of combined lifestyle index with migraine prevalence and headache frequency in Hong Kong Chinese women (October 2019–December 2020)

Baseline data from a cohort study named the Migraine Exposures and Cardiovascular Health in Hong Kong Chinese Women (MECH-HK) were used for this cross-sectional study. The MECH-HK cohort study enrolled 4221 women aged ≥30 years at baseline (October 2019–December 2020). The exclusion criteria were: (1) having headaches other than migraine and (2) having incomplete data on lifestyles, migraine, or other covariates (Fig. 1). The study was approved by the Human Subjects Research Ethics Committee at the Hong Kong Polytechnic University (Ref.: HSEARS20171229004). All participants provided written informed consent.

### Definition and assessment of the combined lifestyle index

The CLI included eight lifestyles: smoking, physical activity, sleep, stress, fatigue, diet, BMI, and alcohol. The selection of these lifestyle factors was based on prior research that investigated the relationship between lifestyle scores/indices and migraine [19, 20], as well as those reviews focusing on migraine-related lifestyle factors [3, 11, 27, 28]. For example, a Norway study incorporated physical activity, smoking, and weight in the lifestyle score [19]. Another German study considered smoking, physical activity, alcohol, and BMI [20]. To ensure comparability with these studies, we first included smoking, physical activity, alcohol, and BMI in our CLI. Next, we found that one review highlighted the association between stress and migraine attack onset [27]. Another review indicated that sleep disorders was significantly related to migraine [3]. Furthermore, several reviews reported relationships of not only individual dietary factors but also dietary patterns with migraine [3, 11, 27, 28]. Moreover, a review reported that premonitory symptoms such as neck pain, fatigue, and sensitivity to lights, sounds, or odors could imitate triggers for migraine [28]. In light of these findings and considering the data available in our database, we included four additional lifestyle factors, namely sleep, stress, fatigue, and diet, into our CLI.

In the CLI, each lifestyle factor was classified as either healthy (1 point) or unhealthy (0 point). The CLI was the sum of these points with a range of 0–8 points (higher scores indicating better health) (Table 1). Specifically, face-to-face questionnaires was used to collect smoking data. In this study, a healthy status was defined as never smoking because a cohort study showed that smokers had a higher migraine incidence than never smokers [4]. The International Physical Activity Questionnaire Short Form was applied to assess physical activity [29]. According to the World Health Organization (WHO) guideline, participants with a ≥ 150 min/week of moderate physical activity or a ≥ 75 min/week of vigorous physical activity were classified as healthy [30]. The evaluation of sleep utilized the Chinese version of the Pittsburgh Sleep Quality Index (PSQI) which comprised seven sections [31]. Each section was allocated a score ranging 0–3, contributing to a total score spanning 0–21, where higher scores denoted poorer sleep quality [31]. In this study, a healthy sleep was regarded as a PSQI score of ≤5 because previous research defined poor sleep quality as a PSQI score of > 5 [31].

**Table 1:** The components and criteria of the combined lifestyle index in Hong Kong Chinese women (October 2019–December 2020)

Component	Score
Smoking	
Never smoker	1
Current or former smoker	0
Physical activity	
Moderate physical activity ≥150 min/week or vigorous physical activity ≥75 min/week	1
Moderate physical activity < 150 min/week and vigorous physical activity < 75 min/week	0
Sleep	
Pittsburgh Sleep Quality Index ≤5	1
Pittsburgh Sleep Quality Index > 5	0
Stress	
Perceived Stress Scale-14 < 25	1
Perceived Stress Scale-14 ≥ 25	0
Fatigue	
<the median of an 11-degree self-perceived fatigue scale	1
≥the median of an 11-degree self-perceived fatigue scale	0
Diet	
≥the median of a healthy diet index	1
<the median of a healthy diet index	0
Body mass index	
≥18.5 and < 23 kg/m^2^	1
<18.5 or ≥ 23 kg/m^2^	0
Alcohol	
Never drinker	1
Current or former drinker	0

Additionally, the Chinese version of the 14-item Perceived Stress Scale (PSS-14) was employed to measure stress [32]. Each item’s frequency was assessed using a 5-point scale, ranging from “never” (0 point) to “very often” (4 point) [32]. The overall PSS-14 was calculated by summing these points, resulting in a range of 0–56, with higher scores correlating to a higher stress perception [32]. In this study, a healthy stress level was defined as a PSS-14 score of < 25, aligning with a previous Chinese study [33]. Furthermore, fatigue data were collected using an 11-degree self-perceived fatigue scale, spanning from 0 (no fatigue) to 10 (the most severe fatigue). Women scoring below the median value of the scale were categorized as healthy.

For the diet, based on the recommendation of the Centre for Health Protection of the Department of Health of Hong Kong, trained research assistants asked subjects regarding their intake frequency of 11 food categories over the past month [34]. These categories included fruits, vegetables, soy-based products, dairy products, fish (excluding salty fish), seafood, meats, eggs, cakes, processed meats, and pickled vegetables. The intake frequencies were “never”, “< 1 time/month”, “1–3 times/month”, “1–3 times/week”, “4–6 times/week”, and “every day”. Similar with a previous Chinese study, a healthy diet index was formulated based on these data [35]. Specifically, in this study, we first performed univariable analyses to explore the associations between the intake frequency of each food category and migraine. Food categories with significantly inverse associations with migraine (fruits, vegetables, and fish) were considered as healthy foods, and were awarded scores of 1–6 for more frequent intake. Conversely, food categories with significantly positive relationships with migraine (dairy products, cakes, processed meats, and pickled vegetables) were regarded as unhealthy foods, and were assigned scores of 6–1 for more frequent consumption. The healthy diet index was computed by summing these scores, resulting in a range of 7–42, with higher scores indicative of a healthier dietary pattern. The associations between the 11 food categories and migraine are shown in the Additional file 1. In the CLI, a healthy diet was defined as ≥ the median value of the healthy diet index.

Moreover, height was assessed using a stadiometer when participants stood without footwear. Weight was evaluated using the Inbody 270 body composition measurement machine, with participants removing heavy clothing and accessories. BMI was computed as weight (kg)/height^2 (m^2). In this study, a healthy BMI was regarded as ≥18.5 and < 23 kg/m^2 based on the WHO recommendation for Asia-Pacific populations [25]. The rationale of this cut-off value was that a meta-analysis indicated that both underweight individuals and obese women had an escalated migraine risk than those with normal weight [7]. Furthermore, alcohol data was obtained by asking the drinking frequency. In this research, being never drinker was considered as healthy, as previous studies showed alcohol was a common trigger for migraine [11].

### Assessment of migraine and other covariates

The diagnosis of migraine started with a questionnaire querying subjects about their headache experiences within the past year. Those who answered “Yes” were directed to complete the ID Migraine™, a rapid screening tool designed for identifying migraine [36, 37]. Participants responding “No” for headache experiences were asked about any prior doctor-diagnosed migraine. Individuals either screening positive with the ID Migraine™ or having a previous migraine diagnosis were considered potential migraineurs. Those with negative ID Migraine™ results or lacking a prior migraine diagnosis underwent an additional evaluation for lifetime migraine symptoms, including photophobia, nausea with headaches, or visual disturbances like blurred vision or flashing lights preceding a headache. Individuals reporting these symptoms were also categorized as potential migraineurs. Conversely, those without these symptoms were classified as no headache. Subsequently, all participants identified as potential migraineurs underwent assessment by a neurologist using the International Classification of Headache Disorders 3rd edition [1]. They were then categorized as having migraine, probable migraine, non-migraine headache, or no headache. For women with migraine or probable migraine, data on migraine attack frequency (attacks/month) were also collected.

Furthermore, face-to-face interviews were conducted to collect the information on additional covariates. These encompassed socioeconomic status (age, marital status, living condition, education, family income, and employment status), women’s health (menstrual age and menopausal status), and medical history.

### Statistical analysis

The baseline characteristics were shown as mean ± standard deviation (SD) for continuous variables and number (percentage) for categorical variables. Normal distribution of all continuous variables was confirmed through Q-Q plots. Based on the sample size of each point category, the CLI was divided into five groups (0–3, 4, 5, 6, and 7–8 points). ANOVA and Chi-square tests were utilized to evaluate the discrepancies in baseline characteristics among the five CLI groups for continuous and categorical variables, respectively.

Univariable and multivariable logistic regression analyses were performed to compute the odds ratios (ORs) and 95% confidence intervals (Cis) for the association between CLI and migraine (complete or probable migraine). In multivariable analyses, three models were utilized with different adjustments: (1) Model I: age; (2) Model II: age, marital status, living condition, education, family income, employment status, menstrual age, and menopause; and (3) Model III: variables in Model II, hypertension, diabetes, hyperlipidaemia, myocardial infarction, stroke, and cancer. Additionally, all analyses were performed for each CLI component individually. In multivariable analyses of each component, the other components were further adjusted. Furthermore, among women with migraine, these analyses were performed to explore the relationship of CLI and its components with migraine attack frequency.

Moreover, several sensitivity analyses were conducted. First, leave-one-out analyses were performed by excluding each component one by one. Second, the lifestyles that exhibited no individual association with migraine were combined as the CLI-weak components. All analyses were then conducted again based on the new CLI. Third, subgroup analyses were carried out based on complete migraine and probable migraine.

Statistical analyses were performed using SPSS 24.0 (SPSS, Inc., New York, USA). Significance levels were established at a two-sided *p* < 0.05.

## Results

### Basic characteristics of participants

Table 2 presents the basic characteristics across the five CLI groups. A total of 3510 women were included, with a mean age of 55.2 ± 9.1 years. In comparison with the lowest CLI group (0–3 points), the highest group (7–8 points) had older ages, higher levels of moderate and vigorous physical activities, lower values of BMI, PSQI, PSS-14, and migraine attack frequency, and escalated healthy diet indices (all *p* < 0.05). Additionally, compared to women with the lowest CLI, those with the highest CLI had an elevated percentage of women with a low family income, women in an unemployed status, menopausal women, never smokers, women reporting minimal or absent fatigue, and never alcohol drinkers (all p < 0.05). No difference was observed for menstrual age, marital status, living condition, hypertension, diabetes, hyperlipidaemia, myocardial infarction, stroke, and cancer.

**Table 2:** Baseline characteristics according to the combined lifestyle index in Hong Kong Chinese women (October 2019–December 2020)

Characteristics ^**a**^	Total subjects	Healthy lifestyle score					*p*
		0–3 points	4 points	5 points	6 points	7–8 points	
N	3510	899	788	776	636	411	
Age, years	55.2 ± 9.1	52.5 ± 9.4	54.9 ± 9.0*	55.8 ± 8.8*	56.8 ± 8.8*	58.3 ± 7.7*	< 0.001
Body mass index, kg/m^2^	23.1 ± 3.6	23.8 ± 4.0	23.4 ± 3.7	23.1 ± 3.5*	22.4 ± 3.1*	21.7 ± 2.4*	< 0.001
Menstrual age, years	12.9 ± 1.7	12.8 ± 1.6	12.9 ± 1.8	12.9 ± 1.7	12.8 ± 1.7	13.0 ± 1.6	0.096
Moderate physical activity, min/week	306.2 ± 313.0	222.5 ± 292.9	287.0 ± 307.7*	333.8 ± 319.1*	360.7 ± 313.0*	390.0 ± 309.3*	< 0.001
Vigorous physical activity, min/week	89.3 ± 162.9	61.8 ± 143.6	81.9 ± 156.9	90.8 ± 166.7*	113.0 ± 174.5*	124.4 ± 176.1*	< 0.001
Pittsburgh Sleep Quality Index	6.1 ± 3.6	8.4 ± 3.2	6.9 ± 3.5*	5.5 ± 3.3*	4.4 ± 2.9*	3.5 ± 2.0*	< 0.001
Perceived Stress Scale-14	24.1 ± 6.0	28.3 ± 4.6	25.2 ± 5.3*	23.3 ± 5.4*	21.0 ± 5.6*	19.4 ± 5.4*	< 0.001
Healthy diet index	31.6 ± 3.4	29.5 ± 2.9	31.4 ± 3.2*	32.1 ± 3.2*	32.8 ± 3.1*	33.8 ± 2.4*	< 0.001
Migraine attack frequency, attacks/month	2.6 ± 3.9	3.2 ± 4.1	2.5 ± 3.5	2.4 ± 4.9	1.6 ± 3.0	0.9 ± 1.0*	0.036
Marital status							0.067
Never married	812 (23.1%)	232 (25.8%)	182 (23.1%)	174 (22.4%)	138 (21.7%)	86 (20.9%)	
Divorce, separation, or widowhood	441 (12.6%)	112 (12.5%)	96 (12.2%)	92 (11.9%)	71 (11.2%)	70 (17.0%)	
Married or cohabiting	2257 (64.3%)	555 (61.7%)	510 (64.7%)	510 (65.7%)	427 (67.1%)	255 (62.0%)	
Living condition							0.147
Living with spouse, parents, or children	683 (19.5%)	183 (20.4%)	151 (19.2%)	142 (18.3%)	111 (17.5%)	96 (23.4%)	
Others	2827 (80.5%)	716 (79.6%)	637 (80.8%)	634 (81.7%)	525 (82.5%)	315 (76.6%)	
Educational level							< 0.001
Primary school or lower	272 (7.7%)	36 (4.0%)	58* (7.4%)	71* (9.1%)	61* (9.6%)	46* (11.2%)	
Secondary school or pre-college	2133 (60.8%)	538 (59.8%)	479 (60.8%)	493 (63.5%)	377 (59.3%)	246 (59.9%)	
College or higher	1105 (31.5%)	325 (36.2%)	251* (31.9%)	212b (27.3%)	198* (31.1%)	119* (29.0%)	
Family income, HKD/month							0.007
≤14,000	1189 (33.9%)	273 (30.4%)	254 (32.2%)	262* (33.8%)	229* (36.0%)	171* (41.6%)	
>14,000 and ≤ 35,000	1441 (41.1%)	386 (42.9%)	321 (40.7%)	325 (41.9%)	250 (39.3%)	159 (38.7%)	
>35,000	880 (25.1%)	240 (26.7%)	213 (27.0%)	189 (24.4%)	157 (24.7%)	81 (19.7%)	
Employed	1838 (52.4%)	566 (63.0%)	430* (54.6%)	379* (48.8%)	295* (46.4%)	168* (40.9%)	< 0.001
Menopause	2534 (72.2%)	558 (62.1%)	559* (70.9%)	579* (74.6%)	495* (77.8%)	343* (83.5%)	< 0.001
Hypertension	423 (12.1%)	94 (10.5%)	101 (12.8%)	97 (12.5%)	82 (12.9%)	49 (11.9%)	0.529
Diabetes	350 (10.0%)	106 (11.8%)	75 (9.5%)	75 (9.7%)	56 (8.8%)	38 (9.2%)	0.310
Hyperlipidaemia	715 (20.4%)	183 (20.4%)	168 (21.3%)	154 (19.8%)	132 (20.8%)	78 (19.0%)	0.891
Myocardial infarction	9 (0.3%)	3 (0.3%)	2 (0.3%)	4 (0.5%)	0 (0.0%)	0 (0.0%)	0.294
Stroke	8 (0.2%)	1 (0.1%)	2 (0.3%)	0 (0.0%)	3 (0.5%)	2 (0.5%)	0.267
Cancer	181 (5.2%)	31 (3.4%)	47 (6.0%)	44 (5.7%)	34 (5.3%)	25 (6.1%)	0.107
Never smoker	3357 (95.6%)	828 (92.1%)	749* (95.1%)	760* (97.9%)	615* (96.7%)	405* (98.5%)	< 0.001
No or low fatigue ^b^	1666 (47.5%)	95 (10.6%)	264* (33.5%)	419* (54.0%)	505* (79.4%)	383* (93.2%)	< 0.001
Never alcohol drinker	1388 (39.5%)	162 (18.0%)	241* (30.6%)	359* (46.3%)	320* (50.3%)	306* (74.5%)	< 0.001

^*^p < 0.05 compared to the 0–3-point group

^a^Continuous and categorical variables were shown as mean ± standard deviation and number (percentage), respectively

^b^No or low fatigue was defined as scoring below or equal to the median value of an 11-degree self-perceived fatigue scale

### Relationships of combined lifestyle index with migraine

The association between CLI and migraine is shown in Table 3. Among the 3510 women, 357 had migraine (10.2%), identified as 230 complete migraineurs and 127 probable migraineurs. The prevalence of migraine in the 0–3-point, 4-point, 5-point, 6-point, and 7–8-point groups was 18.0% (162/899), 10.9% (86/788), 6.6% (51/776), 6.0% (38/636), and 4.9% (20/411), respectively. In the most-adjusted model (Model III), compared to the 0–3-point group, the ORs (95% Cis) for the 4-point, 5-point, 6-point, and 7–8-point groups were 0.57 (0.43–0.75), 0.33 (0.24–0.46), 0.30 (0.21–0.44), and 0.25 (0.15–0.41), respectively (all *p* < 0.001). The inverse relationships were consistent in univariable analysis, Model I, and Model II.

**Table 3:** Relationships between combined lifestyle index and migraine in Hong Kong Chinese women (October 2019–December 2020)

Exposure	N_**case**_	N_**total**_	Prevalence	Univariable analysis		Model I ^**a**^		Model II ^**b**^		Model III ^**c**^	
				OR (95% CI)	*p*	OR (95% CI)	*p*	OR (95% CI)	*p*	OR (95% CI)	*p*
Overall	357	3510	10.2%								
CLI											
0–3 points	162	899	18.0%	Referent		Referent		Referent		Referent	
4 points	86	788	10.9%	0.56 (0.42–0.74)	< 0.001	0.58 (0.44–0.77)	< 0.001	0.57 (0.43–0.76)	< 0.001	0.57 (0.43–0.75)	< 0.001
5 points	51	776	6.6%	0.32 (0.23–0.45)	< 0.001	0.34 (0.24–0.47)	< 0.001	0.33 (0.24–0.47)	< 0.001	0.33 (0.24–0.46)	< 0.001
6 points	38	636	6.0%	0.29 (0.20–0.42)	< 0.001	0.31 (0.21–0.45)	< 0.001	0.30 (0.21–0.44)	< 0.001	0.30 (0.21–0.44)	< 0.001
7–8 points	20	411	4.9%	0.23 (0.14–0.38)	< 0.001	0.25 (0.16–0.41)	< 0.001	0.25 (0.15–0.41)	< 0.001	0.25 (0.15–0.41)	< 0.001
Smoking											
0 point	24	153	15.7%	Referent		Referent		Referent		Referent	
1 point	333	3357	9.9%	0.59 (0.38–0.93)	0.022	0.73 (0.46–1.17)	0.193	0.71 (0.44–1.15)	0.163	0.69 (0.43–1.11)	0.129
Physical activity											
0 point	113	944	12.0%	Referent		Referent		Referent		Referent	
1 point	244	2566	9.5%	0.77 (0.61–0.98)	0.033	0.93 (0.72–1.20)	0.570	0.94 (0.72–1.22)	0.635	0.92 (0.71–1.19)	0.525
Sleep											
0 point	254	1789	14.2%	Referent		Referent		Referent		Referent	
1 point	103	1721	6.0%	0.38 (0.30–0.49)	< 0.001	0.49 (0.38–0.64)	< 0.001	0.49 (0.38–0.64)	< 0.001	0.50 (0.38–0.65)	< 0.001
Stress											
0 point	237	1832	12.9%	Referent		Referent		Referent		Referent	
1 point	120	1678	7.2%	0.52 (0.41–0.65)	< 0.001	0.75 (0.59–0.96)	0.023	0.74 (0.58–0.95)	0.016	0.74 (0.58–0.95)	0.019
Fatigue											
0 point	253	1844	13.7%	Referent		Referent		Referent		Referent	
1 point	104	1666	6.2%	0.42 (0.33–0.53)	< 0.001	0.62 (0.48–0.81)	< 0.001	0.63 (0.48–0.82)	0.001	0.62 (0.48–0.81)	< 0.001
Diet											
0 point	219	1692	12.9%	Referent		Referent		Referent		Referent	
1 point	138	1818	7.6%	0.55 (0.44–0.69)	< 0.001	0.66 (0.52–0.83)	< 0.001	0.66 (0.52–0.83)	< 0.001	0.65 (0.52–0.83)	< 0.001
Body mass index											
0 point	182	1793	10.2%	Referent		Referent		Referent		Referent	
1 point	175	1717	10.2%	1.00 (0.81–1.25)	0.967	1.00 (0.80–1.26)	0.980	0.99 (0.79–1.24)	0.925	0.98 (0.78–1.23)	0.872
Alcohol											
0 point	243	2122	11.5%	Referent		Referent		Referent		Referent	
1 point	114	1388	8.2%	0.69 (0.55–0.87)	0.002	0.80 (0.63–1.02)	0.070	0.81 (0.63–1.03)	0.082	0.81 (0.63–1.03)	0.088
CLI (no smoking)											
0–2 points	161	886	18.2%	Referent		Referent		Referent		Referent	
3 points	85	783	10.9%	0.55 (0.41–0.73)	< 0.001	0.57 (0.43–0.75)	< 0.001	0.56 (0.42–0.75)	< 0.001	0.56 (0.42–0.74)	< 0.001
4 points	53	790	6.7%	0.32 (0.23–0.45)	< 0.001	0.34 (0.24–0.47)	< 0.001	0.34 (0.24–0.47)	< 0.001	0.34 (0.24–0.47)	< 0.001
5 points	38	633	6.0%	0.29 (0.20–0.42)	< 0.001	0.31 (0.21–0.45)	< 0.001	0.30 (0.21–0.44)	< 0.001	0.30 (0.21–0.44)	< 0.001
6–7 points	20	418	4.8%	0.23 (0.14–0.37)	< 0.001	0.25 (0.15–0.40)	< 0.001	0.24 (0.15–0.39)	< 0.001	0.24 (0.15–0.39)	< 0.001
CLI (no physical activity)											
0–2 points	129	692	18.6%	Referent		Referent		Referent		Referent	
3 points	98	759	12.9%	0.65 (0.49–0.86)	0.003	0.66 (0.50–0.88)	0.005	0.66 (0.49–0.88)	0.004	0.65 (0.49–0.87)	0.004
4 points	64	845	7.6%	0.36 (0.26–0.49)	< 0.001	0.37 (0.27–0.51)	< 0.001	0.37 (0.27–0.51)	< 0.001	0.37 (0.27–0.51)	< 0.001
5 points	42	723	5.8%	0.27 (0.19–0.39)	< 0.001	0.28 (0.20–0.41)	< 0.001	0.28 (0.19–0.40)	< 0.001	0.28 (0.19–0.41)	< 0.001
6–7 points	24	491	4.9%	0.22 (0.14–0.35)	< 0.001	0.24 (0.15–0.38)	< 0.001	0.24 (0.15–0.37)	< 0.001	0.24 (0.15–0.37)	< 0.001
CLI (no sleep)											
0–2 points	89	475	18.7%	Referent		Referent		Referent		Referent	
3 points	93	704	13.2%	0.66 (0.48–0.91)	0.010	0.69 (0.50–0.95)	0.022	0.69 (0.50–0.95)	0.021	0.68 (0.49–0.94)	0.020
4 points	90	958	9.4%	0.45 (0.33–0.62)	< 0.001	0.48 (0.35–0.66)	< 0.001	0.47 (0.34–0.65)	< 0.001	0.47 (0.34–0.65)	< 0.001
5 points	54	807	6.7%	0.31 (0.22–0.45)	< 0.001	0.34 (0.23–0.49)	< 0.001	0.33 (0.23–0.48)	< 0.001	0.33 (0.23–0.48)	< 0.001
6–7 points	31	566	5.5%	0.25 (0.16–0.39)	< 0.001	0.28 (0.18–0.44)	< 0.001	0.27 (0.18–0.43)	< 0.001	0.27 (0.17–0.42)	< 0.001
CLI (no stress)											
0–2 points	92	452	20.4%	Referent		Referent		Referent		Referent	
3 points	107	749	14.3%	0.65 (0.48–0.89)	0.006	0.67 (0.49–0.92)	0.012	0.67 (0.49–0.91)	0.010	0.67 (0.49–0.91)	0.010
4 points	70	901	7.8%	0.33 (0.24–0.46)	< 0.001	0.35 (0.25–0.49)	< 0.001	0.34 (0.24–0.48)	< 0.001	0.34 (0.24–0.48)	< 0.001
5 points	60	846	7.1%	0.30 (0.21–0.42)	< 0.001	0.32 (0.23–0.46)	< 0.001	0.32 (0.22–0.45)	< 0.001	0.31 (0.22–0.45)	< 0.001
6–7 points	28	562	5.0%	0.21 (0.13–0.32)	< 0.001	0.22 (0.14–0.35)	< 0.001	0.22 (0.14–0.35)	< 0.001	0.22 (0.14–0.34)	< 0.001
CLI (no fatigue)											
0–2 points	84	440	19.1%	Referent		Referent		Referent		Referent	
3 points	102	723	14.1%	0.70 (0.51–0.96)	0.025	0.73 (0.53–1.00)	0.050	0.73 (0.53–1.00)	0.050	0.73 (0.53–1.01)	0.054
4 points	87	943	9.2%	0.43 (0.31–0.60)	< 0.001	0.46 (0.33–0.64)	< 0.001	0.46 (0.33–0.63)	< 0.001	0.45 (0.32–0.63)	< 0.001
5 points	56	862	6.5%	0.29 (0.21–0.42)	< 0.001	0.32 (0.22–0.46)	< 0.001	0.32 (0.22–0.46)	< 0.001	0.31 (0.22–0.46)	< 0.001
6–7 points	28	542	5.2%	0.23 (0.15–0.36)	< 0.001	0.26 (0.16–0.40)	< 0.001	0.25 (0.16–0.40)	< 0.001	0.25 (0.16–0.39)	< 0.001
CLI (no diet)											
0–2 points	102	503	20.3%	Referent		Referent		Referent		Referent	
3 points	101	780	12.9%	0.58 (0.43–0.79)	< 0.001	0.60 (0.45–0.82)	0.001	0.61 (0.45–0.82)	0.001	0.60 (0.44–0.82)	0.001
4 points	71	849	8.4%	0.36 (0.26–0.50)	< 0.001	0.38 (0.27–0.52)	< 0.001	0.38 (0.27–0.53)	< 0.001	0.38 (0.27–0.52)	< 0.001
5 points	48	772	6.2%	0.26 (0.18–0.38)	< 0.001	0.28 (0.19–0.40)	< 0.001	0.28 (0.19–0.40)	< 0.001	0.28 (0.19–0.40)	< 0.001
6–7 points	35	606	5.8%	0.24 (0.16–0.36)	< 0.001	0.26 (0.17–0.40)	< 0.001	0.26 (0.17–0.39)	< 0.001	0.26 (0.17–0.39)	< 0.001
CLI (no body mass index)											
0–2 points	116	566	20.5%	Referent		Referent		Referent		Referent	
3 points	91	674	13.5%	0.61 (0.45–0.82)	0.001	0.62 (0.46–0.85)	0.002	0.62 (0.46–0.85)	0.002	0.61 (0.45–0.83)	0.002
4 points	74	845	8.8%	0.37 (0.27–0.51)	< 0.001	0.39 (0.28–0.54)	< 0.001	0.39 (0.28–0.53)	< 0.001	0.39 (0.28–0.53)	< 0.001
5 points	46	760	6.1%	0.25 (0.17–0.36)	< 0.001	0.27 (0.18–0.38)	< 0.001	0.26 (0.18–0.38)	< 0.001	0.26 (0.18–0.37)	< 0.001
6–7 points	30	665	4.5%	0.18 (0.12–0.28)	< 0.001	0.20 (0.13–0.31)	< 0.001	0.20 (0.13–0.30)	< 0.001	0.20 (0.13–0.30)	< 0.001
CLI (no alcohol)											
0–2 points	103	503	20.5%	Referent		Referent		Referent		Referent	
3 points	87	655	13.3%	0.59 (0.44–0.81)	0.001	0.62 (0.45–0.84)	0.002	0.61 (0.44–0.84)	0.002	0.62 (0.45–0.84)	0.003
4 points	90	901	10.0%	0.43 (0.32–0.59)	< 0.001	0.45 (0.33–0.62)	< 0.001	0.45 (0.33–0.61)	< 0.001	0.44 (0.32–0.61)	< 0.001
5 points	37	741	5.0%	0.20 (0.14–0.30)	< 0.001	0.22 (0.15–0.32)	< 0.001	0.21 (0.14–0.32)	< 0.001	0.21 (0.14–0.31)	< 0.001
6–7 points	40	710	5.6%	0.23 (0.16–0.34)	< 0.001	0.25 (0.17–0.38)	< 0.001	0.25 (0.17–0.37)	< 0.001	0.25 (0.17–0.37)	< 0.001
CLI-weak components ^d^											
0–1 points	53	375	14.1%	Referent		Referent		Referent		Referent	
2 points	138	1214	11.4%	0.78 (0.55–1.10)	0.151	0.84 (0.60–1.18)	0.320	0.84 (0.59–1.18)	0.317	0.83 (0.59–1.17)	0.287
3–4 points	166	1921	8.6%	0.57 (0.41–0.80)	0.001	0.65 (0.47–0.92)	0.014	0.65 (0.46–0.92)	0.014	0.63 (0.45–0.89)	0.009

*OR* Odds ratio, *CI* Confidence interval, *CLI* Combined lifestyle index

^a^Model I adjusted for age. In the analyses of each individual component, the other components were further adjusted

^b^Model II adjusted for age, marital status, living condition, educational level, family income, employment status, menstrual age, and menopause. In the analyses of each individual component, the other components were further adjusted

^c^Model III adjusted for variables in Model II, hypertension, diabetes, hyperlipidaemia, myocardial infarction, stroke, and cancer. In the analyses of each individual component, the other components were further adjusted

^d^Four lifestyle factors (smoking, physical activity, body mass index, and alcohol) that were not independently associated with migraine were included in the CLI-weak components

For the analyses of each component, migraine was inversely correlated with the scores of sleep, stress, fatigue, and diet (1 vs. 0 points) (all p < 0.001); but was unrelated to smoking, physical activity, BMI, and alcohol (all *p* > 0.05) (Table 3). Additionally, among women with migraine (*N* = 357), the average migraine attack frequency was 2.6 ± 3.9 attacks/month (Table 2). Per one point increase in CLI was associated with a reduced migraine attack frequency (the most-adjusted β = − 0.55; 95% CI = -0.82, − 0.28; p < 0.001) (Table 4). While the inverse association was only observed for fatigue and BMI (Table 4).

**Table 4:** Relationships between combined lifestyle index and migraine attack frequency (attacks/month) in Hong Kong Chinese women with migraine (*N* = 357) (October 2019–December 2020)

Exposure ^**a**^	Univariable analysis		Model I ^**b**^		Model II ^**c**^		Model III ^**d**^	
	β (95% CI)	*p*	β (95% CI)	*p*	β (95% CI)	*p*	β (95% CI)	*p*
Combined lifestyle index	− 0.46 (− 0.72, − 0.20)	< 0.001	− 0.60 (− 0.94, − 0.27)	< 0.001	− 0.61 (− 0.95, − 0.27)	0.001	− 0.55 (− 0.82, − 0.28)	< 0.001
Smoking	−0.15 (− 1.84, 1.55)	0.864	− 0.37 (− 2.07, 1.34)	0.672	− 0.39 (− 2.13, 1.34)	0.655	− 0.32 (− 2.06, 1.42)	0.718
Physical activity	0.08 (− 0.81, 0.97)	0.862	− 0.19 (− 1.12, 0.74)	0.686	− 0.40 (− 1.35, 0.56)	0.418	−0.51 (− 1.47, 0.46)	0.305
Sleep	−0.69 (− 1.71, 0.33)	0.185	− 0.74 (− 1.76, 0.28)	0.156	− 0.78 (− 1.81, 0.25)	0.138	−0.74 (− 1.78, 0.29)	0.159
Stress	−0.57 (− 1.52, 0.37)	0.234	− 0.56 (− 1.50, 0.38)	0.242	− 0.52 (− 1.46, 0.43)	0.283	−0.49 (− 1.44, 0.46)	0.311
Fatigue	−1.15 (−2.15, − 0.14)	0.026	−1.20 (− 2.21, − 0.20)	0.019	−1.13 (− 2.14, − 0.11)	0.030	−1.12 (− 2.14, − 0.09)	0.033
Diet	0.32 (− 0.52, 1.17)	0.450	0.25 (− 0.59, 1.09)	0.561	0.13 (− 0.74, 0.99)	0.772	0.13 (− 0.76, 1.02)	0.774
Body mass index	−0.96 (− 1.77, − 0.14)	0.022	−0.88 (− 1.70, − 0.07)	0.033	−0.82 (− 1.64, 0.00)	0.051	− 0.84 (− 1.67, − 0.02)	0.046
Alcohol	0.10 (− 0.79, 0.99)	0.828	0.05 (− 0.84, 0.94)	0.907	0.07 (− 0.83, 0.96)	0.881	0.12 (− 0.78, 1.03)	0.789

*OR* Odds ratio, *CI* Confidence interval

^a^The β of per point increase in the combined lifestyle index and factors

^b^Model I adjusted for age. In the analyses of each individual component, the other components were further adjusted

^c^Model II adjusted for age, marital status, living condition, educational level, family income, employment status, menstrual age, and menopause. In the analyses of each individual component, the other components were further adjusted

^d^Model III adjusted for variables in Model II, hypertension, diabetes, hyperlipidaemia, myocardial infarction, stroke, and cancer. In the analyses of each individual component, the other components were further adjusted

### Sensitivity analysis

In leave-one-out analyses, CLI was consistently inversely related to migraine after excluding each component one at a time (Table 3). Additionally, when combining the four lifestyles that were not individually associated with migraine, the new CLI was still inversely related to migraine, with the most-adjusted OR (95% CI) of 0.63 (0.45–0.89) (*p* = 0.009) (the highest vs. lowest groups) (Table 3). Moreover, in subgroup analyses, similar results were observed for complete migraine (Additional file 2) and probable migraine (Additional file 3).

## Discussion

### Summary of findings

This cross-sectional study found an inverse association between an 8-item CLI and migraine prevalence in Hong Kong Chinese women. The results were consistent in leave-one-out analyses. Among the eight components, migraine was significantly associated with sleep, stress, fatigue, and diet; but was not related to smoking, physical activity, BMI, and alcohol. Furthermore, combining the four lifestyles that showed no individual association with migraine still maintained the inverse relationship between CLI and migraine. Subgroup analyses based on migraine subtypes also generated similar results. Among women with migraine, CLI was inversely associated with migraine attack frequency.

### Comparisons with previous research and explanations

There were limited studies exploring the associations between combined lifestyles and migraine. A Norway cross-sectional study of 5588 students (12–19 years) found that, compared to participants with all three health lifestyles (high physical activity, non-smoking, and normal weight), subjects with two, one, and zero lifestyles had increased migraine prevalence, with ORs (95% CIs) of 1.5 (1.2–1.9), 2.1 (1.5–2.8), and 3.7 (1.9–7.1), respectively [19]. Conversely, another German cross-sectional study of 6309 participants (35–75 years) showed that a 4-item health index (smoking, physical activity, alcohol, and BMI) was not associated with migraine [20]. There are several possible reasons for the different results. First, the two studies recruited participants with different ages (12–19 vs. 35–75 years). Second, the difference in lifestyle components might contribute to the discrepancies. Third, the sample sizes were different between the studies. Specifically, although the German study had 6309 participants, it was actually performed separately by three databases [20]. Therefore, the sample size of each dataset might not be sufficient.

Unlike the limited studies focusing on combined lifestyles, many prior studies have investigated the relationships between individual lifestyles and migraine. For example, an 11-year cohort research in Norway found that, among 15,276 individuals without a baseline headache, the migraine incidence was higher in smokers than in never smokers, with a relative risk (RR) (95% CI) of 1.30 (1.11–1.52) [4]. Meanwhile, that study also indicated that, compared to subjects being physically inactive, those with light (1–3 h/week) and vigorous physical activity (1–2 h/week) had reduced migraine incidence, with RRs (95% CIs) of 0.78 (0.62–0.99) and 0.71 (0.54–0.94), respectively [4]. Likewise, a Swedish cross-sectional study of 43,770 subjects (18–79 years) revealed that smoking and physical inactivity were positively associated with migraine [38]. However, a German cross-sectional study with 6309 subjects reported no associations of smoking and physical activity with migraine [20]. While the relatively small sample size might partially explain the difference.

Regarding sleep, a meta-analysis of 23 case-control studies showed that the PSQI score of adults with migraine was higher than that of controls [5]. Additionally, a randomized controlled trial (RCT) of 31 US adults with chronic migraine and insomnia indicated that the insomnia cognitive-behavioral therapy group had a lower headache event rate than the control group (38.8% vs. 48.1%; OR = 0.40; 95% CI = 0.17–0.91; *p* = 0.028) [39]. Another RCT of 43 US women with transformed migraine also found that the behavioral sleep modification could reduce headache frequency and intensity [40]. For stress, a meta-analysis of 2 RCTs with 43 patients found that mindfulness-based stress reduction could decrease migraine pain intensity (standardized mean difference = − 0.84; 95% CI = -1.48 to − 0.19; *p* = 0.01) [6]. Additionally, a prospective cohort study of 1125 US individuals with episodic migraine suggested that tiredness/fatigue was positively correlated with migraine [8]. Moreover, a meta-analysis of 11 studies found that, compared to normal-weight subjects, underweight people and obese women had an increased migraine risk, with ORs (95% CIs) of 1.21 (1.07–1.37) (*p* = 0.002) and 1.44 (1.05–1.97) (*p* = 0.023), respectively [7].

Meanwhile, previous studies identified several dietary factors and patterns associated with migraine. A cross-sectional study of 25,755 US women indicated that, compared to participants with migraine without aura, those with migraine with aura had a low intake of chocolate, cheese, ice cream, hot dogs, and processed meats [10]. In an Iran cross-sectional study with 224 women with migraine (20–50 years), inverse associations were observed between the Mediterranean diet and migraine headache index score, headache frequency, headache duration, and headache impact test-6 [12]. Another Iran cross-sectional study of 285 women with migraine also found negative relationships of the Dietary Approaches to Stop Hypertension (DASH) diet with migraine index score and attack frequency [13]. For alcohol, a recent meta-analysis of 19 studies with 126,173 participants showed that alcohol drinkers had a lower migraine risk than non-drinkers (RR = 0.71; 95% CI = 0.57–0.89) [41]. One potential reason for the inverse alcohol-migraine relationship could be that patients with migraine might abstain from alcohol due to its capacity to trigger headache [38].

The advantage of combining lifestyle factors lies in considering their potential interactions. Individuals often adopt multiple lifestyles simultaneously, and these factors may interact, yielding different associations with migraine compared to individual analyses. Our aim was to evaluate not only individual lifestyle factors but also the overall lifestyle pattern in relation to migraine. Our results showed that, compared to the lowest CLI group, the ORs of the other four groups seemed to be lower than those of significant individual lifestyle factors. Although the differences in the ORs did not achieve statistical significance. Additionally, when we devised a new CLI that incorporated the four components exhibiting no individual association with migraine in their respective analyses, the new index consistently exhibited an inverse relationship with migraine. These findings underscored the importance of a comprehensive healthy lifestyle pattern. However, combining lifestyles does not mean neglecting individual factors. Hence, we presented results for both overall CLI and each component individually. Moreover, to discern whether any individual lifestyle factor exerted a significant influence on the results of the CLI, we conducted leave-one-out analyses by excluding each item from the index one at a time. The results of leave-one-out analyses were consistent with the main results, suggesting the robustness of the index.

### Mechanism

The correlations between lifestyles and migraine could be partially explained by some mechanisms. For example, the analgesic property of tobacco might influence the central nervous system and then cause headache [4]. However, it is also possible that patients with migraine might smoke more than non-headache subjects due to headaches [4]. The mechanisms of physical activity might involve elevated plasma levels of beta-endorphin, endocannabinoids, and brain-derived neurotrophic factor [4, 42]. Additionally, physical activity might interact with other lifestyles like BMI [4, 42]. By contrast, it is also reported that those with migraine might avoid physical activity [4, 42].

Furthermore, the sleep-migraine relationship might be associated with their shared brain regions [43]. Rapid eye movement sleep was regulated by cells in certain brain regions, such as the ventrolateral periaqueductal gray (vPAG) which was supplied by orexinergic inputs from the hypothalamus [43]. However, the vPAG also exerted an inhibitory impact on the nociception in the trigeminal nucleus caudalis (TNC), the principal region in the brainstem accountable for the perception of head pain [43]. Hence, disturbances in sleep might interfere with the signaling from hypothalamus to vPAG, consequently impacting its ability to inhibit pain perception in the TNC, thereby leading to headache attack [43]. Conversely, migraine might also lead to poor sleep via these shared brain regions [43].

Additionally, stress could directly affect the autonomic nervous and neuroendocrine systems, potentially leading to a sensitization of nociceptors [44]. Prolonged exposure to stress might imped the brain’s capacity to maintain allostasis [44]. Furthermore, stress might also indirectly cause headaches by contributing to other unhealthy lifestyles, such as poor diet, sleep, and fatigue [44]. In contrast, subjects with migraine were also reported to experience more stress than non-headache individuals [44].

For diet, the food categories included in this study might partially explain the inverse correlation. These food categories contained high amounts of fiber, vitamin B, vitamin C, coenzyme Q10, and magnesium; and low amounts of fat and sodium [13, 45]. Some of these constituents exhibited anti-inflammatory and antioxidant properties, potentially inhibiting the generation of inflammatory cytokines and mitigating neurogenic inflammation associated with migraine [13]. For example, high-fiber diets were linked to inflammation reduction by impeding glucose absorption and modifying gut microflora [45]. The low levels of riboflavin, coenzyme Q10, and magnesium in migraineurs might contribute to the pathophysiology, as these nutrients were essential for energy generation within mitochondria [13]. Meanwhile, magnesium could prevent migraine by blocking N-Methyl-D-aspartate receptors, curtailing serotonin-dependent vascular spasms, and hindering platelet aggregation [13].

For obesity, adipose tissues can release proinflammatory cytokines (e.g., tumor necrosis factor alpha, interleukin-1, interleukin-6, and adiponectin), which could activate the nitric oxide pathway in the brain and then cause headaches [7]. For underweight, factors like psychiatric comorbidities (e.g., anxiety, depression, stress, and fatigue) might serve as potential contributors [7]. Furthermore, potential mechanisms behind alcohol-induced migraine involved the vasodilation of cerebral blood vessels after drinking and the receptors located in the cortex or brainstem [41].

Overall, there were some common mechanisms for the association between lifestyle factors and migraine, involving the influence of both brain function and structure, as well as eliciting inflammatory responses. Additionally, these lifestyles demonstrated significant interconnections, potentially culminating in a synergistic impact on migraine. On the contrary, individuals with migraine might also have some unhealthy lifestyles due to headaches.

### Strengths and limitations

Although this might be the first cross-sectional study to explore the correlation between CLI and migraine in Chinese people, there are several limitations. First, due to its cross-sectional nature, this study cannot establish causal associations. Further cohort studies involving the CLI or RCTs based on some behavior change interventions should be performed. For instance, Robblee J et al. proposed that primary care physicians can help patients with migraine reduce attack likelihood and symptom severity through lifestyle counselling related to the SEEDS intervention (sleep, exercise, eat, diary, and stress) [46]. The SEEDS program included adherence to standard sleep hygiene for optimal sleep quantity and quality (S); engagement of 30–60 minutes of physical activity 3–5 times/week (E); regular and nutritious meals with controlled caffeine intake (E); the utilization of a headache diary for the follow-up of headache (D); and stress management such as cognitive behavioral therapy, mindfulness, relaxation, or biofeedback (S) [46]. Thus, the application of our CLI in RCTs may be similar to the SEEDS program, which includes a comprehensive set of interventions targeting each lifestyle factor within the CLI, along with specific methods for the management of migraine such as a headache diary.

Second, recall bias might exist due to the use of questionnaires. Nevertheless, we mitigated this bias by using face-to-face interviews rather than self-administered questionnaires. Third, the definitions of “healthy” for some lifestyle factors were relatively arbitrary, which introduced the inconsistencies in defining “healthy” across CLI components. For instance, a healthy diet was delineated as ≥ the median value of a healthy diet index. Whereas the dietary data only contained the intake frequency of several food items. This method, however, lacked consideration for actual intake quantities and overlooked other key food categories like whole grains. Future studies should incorporate more dietary data. In addition, a healthy status of fatigue was established as below the median value of an 11-degree self-perceived fatigue scale, rather than some structured tools like the fatigue severity scale. The two arbitrary definitions were different with the definitions of other components, which were derived from previous studies or recognized standards. Therefore, further studies are recommended to include more detailed data on diet and fatigue with the use of some systematic tools.

Fourth, in this study, BMI was dichotomously classified as falling within the range of 18.5–23 kg/m^2 or not, thus grouping underweight and overweight individuals together. This classification approach raises concerns regarding potential misclassification, given that the associations of migraine with underweight, normal weight, and overweight might be different [7]. Whereas the selection of this threshold was based on a meta-analysis, which demonstrated that both underweight individuals and obese women exhibited an increased risk of migraine compared to those within the normal weight range [7]. Thus, employing this cut-off value for BMI may have minimal misclassification biases.

Finally, the study exclusively involved Hong Kong Chinese women. The main advantage of such a target population was the reduction of potential confounders associated with sex-specific physiological and hormonal differences, since migraine prevalence has been observed to be higher in women than men [2]. However, it should be noted that, by limiting the population to Hong Kong Chinese women, the generalizability of the findings to men and other ethnicities is limited. Moreover, by only focusing on women, potential interactions between sex and other variables that related to the lifestyle-migraine association might be overlooked. Future studies that include both sexes are needed to provide a more comprehensive understanding of the relationship between lifestyle and migraine.

## Conclusion

In summary, this cross-sectional investigation showed an inverse correlation between an 8-item CLI-encompassing smoking, physical activity, sleep, stress, fatigue, diet, BMI, and alcohol-and the prevalence of migraine among Hong Kong Chinese women. The inverse association remained robust across leave-one-out and subgroup analyses. Among the eight lifestyle factors, migraine was significantly associated with sleep, stress, fatigue, and diet; but was not related to smoking, physical activity, BMI, and alcohol. Furthermore, among women with migraine, CLI was inversely associated with migraine attack frequency. However, the inherent limitation of the cross-sectional design prohibits the establishment of causal associations based on the current findings. It might be plausible that adhering to a healthy lifestyle pattern could potentially contribute to the prevention of migraine attacks. Conversely, it is equally conceivable that women experiencing migraine might demonstrate an unhealthier lifestyle pattern compared to women without headaches. Further cohort studies or RCTs are needed to confirm our findings. Additionally, future investigations are advised to incorporate more detailed data on diet and fatigue, while also encompassing both women and men to offer a more comprehensive understanding of the relationship between lifestyle factors and migraine.

### Supplementary Information

**Additional file 1.** Relationships between 11 dietary habits and migraine in Hong Kong Chinese women (October 2019–December 2020).

**Additional file 2.** Relationships between combined lifestyle index and complete migraine in Hong Kong Chinese women (October 2019–December 2020).

**Additional file 3.** Relationships between combined lifestyle index and probable migraine in Hong Kong Chinese women (October 2019–December 2020).

## Data Availability

The datasets used and/or analysed during the current study are available from the corresponding author on reasonable request.

## Abbreviations

BMI: Body mass index
CI: Confidence interval
CLI: Combined lifestyle index
MECH-HK: Migraine Exposures and Cardiovascular Health in Hong Kong Chinese Women
OR: Odds ratio
PSQI: Pittsburgh Sleep Quality Index
PSS-14: 14-item Perceived Stress Scale
RCT: Randomized controlled trial
RR: Relative risk
SD: Standard deviation
TNC: Trigeminal nucleus caudalis
US: United States
vPAG: Ventrolateral periaqueductal gray
