# Supplementary material for: Associations of combined lifestyle index with migraine prevalence and headache frequency: a cross-sectional study from the MECH-HK study
Source: J Headache Pain. 2024 Feb 20;25(1):24. doi: 10.1186/s10194-024-01729-y (PMC10877907; doi:10.1186/s10194-024-01729-y)
Supplement: Supplementary file 1 — Additional file 1. Relationships between 11 dietary habits and migraine in Hong Kong Chinese women (October 2019–December 2020). [file 10194_2024_1729_MOESM1_ESM.docx]

# Additional file 1. Relationships between 11 dietary habits and migraine in Hong Kong Chinese women (October 2019-December 2020)

| **Food and intake frequency** | **N_case_** | **N_total_** | **Prevalence** | **Univariable analysis** | |
| --- | --- | --- | --- | --- | --- |
|  |  |  |  | **OR (95% CI)** | **p** |
| Overall | 357 | 3,510 | 10.2% |  |  |
| Fruits |  |  |  |  |  |
| Never or ≤1-3 times/week | 115 | 886 | 13.0% | Referent |  |
| 4-6 times/week | 94 | 884 | 10.6% | 0.80 (0.60-1.07) | 0.127 |
| Every day | 148 | 1,740 | 8.5% | 0.62 (0.48-0.81) | <0.001 |
| Vegetables |  |  |  |  |  |
| Never or ≤1-3 times/week | 58 | 465 | 12.5% | Referent |  |
| 4-6 times/week | 92 | 826 | 11.1% | 0.88 (0.62-1.25) | 0.473 |
| Every day | 207 | 2,219 | 9.3% | 0.72 (0.53-0.98) | 0.039 |
| Soy-based products |  |  |  |  |  |
| Never or ≤1-3 times/month | 161 | 1,480 | 10.9% | Referent |  |
| 1-3 times/week | 127 | 1,389 | 9.1% | 0.82 (0.65-1.05) | 0.123 |
| ≥4-6 times/week | 69 | 641 | 10.8% | 0.99 (0.73-1.33) | 0.938 |
| Dairy products |  |  |  |  |  |
| Never or <1 times/month | 63 | 730 | 8.6% | Referent |  |
| 1-3 times/month | 97 | 981 | 9.9% | 1.16 (0.83-1.62) | 0.377 |
| ≥1-3 times/week | 197 | 1,799 | 11.0% | 1.30 (1.01-1.75) | 0.048 |
| Fish (excluding salty fish) |  |  |  |  |  |
| Never or ≤1-3 times/month | 133 | 1,052 | 12.6% | Referent |  |
| 1-3 times/week | 163 | 1,564 | 10.4% | 0.80 (0.63-1.03) | 0.079 |
| ≥4-6 times/week | 61 | 894 | 6.8% | 0.51 (0.37-0.69) | <0.001 |
| Seafood |  |  |  |  |  |
| Never or <1 times/month | 74 | 855 | 8.7% | Referent |  |
| 1-3 times/month | 159 | 1,468 | 10.8% | 1.28 (0.96-1.71) | 0.093 |
| ≥1-3 times/week | 124 | 1,187 | 10.4% | 1.23 (0.91-1.67) | 0.178 |
| Meats |  |  |  |  |  |
| Never or ≤1-3 times/week | 121 | 1,138 | 10.6% | Referent |  |
| 4-6 times/week | 106 | 1,066 | 9.9% | 0.93 (0.70-1.22) | 0.595 |
| Every day | 130 | 1,306 | 10.0% | 0.93 (0.72-1.21) | 0.582 |
| Eggs |  |  |  |  |  |
| Never or ≤1-3 times/month | 29 | 367 | 7.9% | Referent |  |
| 1-3 times/week | 131 | 1,289 | 10.2% | 1.32 (0.87-2.01) | 0.197 |
| 4-6 times/week | 112 | 998 | 11.2% | 1.47 (0.96-2.26) | 0.075 |
| Every day | 85 | 856 | 9.9% | 1.28 (0.83-2.00) | 0.265 |
| Cakes |  |  |  |  |  |
| Never | 36 | 415 | 8.7% | Referent |  |
| <1 times/month | 136 | 1,513 | 9.0% | 1.04 (0.71-1.53) | 0.842 |
| 1-3 times/month | 128 | 1,142 | 11.2% | 1.33 (0.90-1.96) | 0.151 |
| ≥1-3 times/week | 57 | 440 | 13.0% | 1.57 (1.01-2.43) | 0.046 |
| Processed meats |  |  |  |  |  |
| Never or <1 times/month | 144 | 1,696 | 8.5% | Referent |  |
| 1-3 times/month | 147 | 1,268 | 11.6% | 1.41 (1.11-1.80) | 0.005 |
| ≥1-3 times/week | 66 | 546 | 12.1% | 1.48 (1.09-2.02) | 0.013 |
| Pickled vegetables |  |  |  |  |  |
| Never | 47 | 530 | 8.9% | Referent |  |
| <1 times/month | 146 | 1,691 | 8.6% | 0.97 (0.69-1.37) | 0.867 |
| ≥1-3 times/month | 164 | 1,289 | 12.7% | 1.50 (1.07-2.11) | 0.020 |

^a^: Some intake frequencies of food categories were combined due to the small sample size of these frequencies. The full list of intake frequency is “never”, “<1 time/month”, “1-3 times/month”, “1-3 times/week”, “4-6 times/week”, and “every day”.
