# Supplementary material for: Associations of combined lifestyle index with migraine prevalence and headache frequency: a cross-sectional study from the MECH-HK study
Source: J Headache Pain. 2024 Feb 20;25(1):24. doi: 10.1186/s10194-024-01729-y (PMC10877907; doi:10.1186/s10194-024-01729-y)
Supplement: Supplementary file 3 — Additional file 3. Relationships between combined lifestyle index and probable migraine in Hong Kong Chinese women (October 2019–December 2020). [file 10194_2024_1729_MOESM3_ESM.docx]

# Additional file 3. Relationships between combined lifestyle index and probable migraine in Hong Kong Chinese women (October 2019-December 2020)

| **Exposure** | **N_case_** | **N_total_** | **Prevalence** | **Univariable analysis** | | **Model I ^a^** | | **Model II ^b^** | | **Model III ^c^** | |
| --- | --- | --- | --- | --- | --- | --- | --- | --- | --- | --- | --- |
|  |  |  |  | **OR (95% CI)** | **p** | **OR (95% CI)** | **p** | **OR (95% CI)** | **p** | **OR (95% CI)** | **p** |
| Overall | 127 | 3,280 | 3.9% |  |  |  |  |  |  |  |  |
| CLI |  |  |  |  |  |  |  |  |  |  |  |
| 0-3 points | 55 | 792 | 6.9% | Referent |  | Referent |  | Referent |  | Referent |  |
| 4 points | 28 | 730 | 3.8% | 0.53 (0.34-0.85) | 0.009 | 0.56 (0.35-0.89) | 0.014 | 0.56 (0.35-0.90) | 0.016 | 0.56 (0.35-0.89) | 0.015 |
| 5 points | 24 | 749 | 3.2% | 0.44 (0.27-0.72) | 0.001 | 0.47 (0.29-0.77) | 0.003 | 0.47 (0.29-0.78) | 0.003 | 0.47 (0.29-0.78) | 0.003 |
| 6 points | 10 | 608 | 1.6% | 0.22 (0.11-0.44) | <0.001 | 0.24 (0.12-0.48) | <0.001 | 0.24 (0.12-0.48) | <0.001 | 0.24 (0.12-0.48) | <0.001 |
| 7-8 points | 10 | 401 | 2.5% | 0.34 (0.17-0.68) | 0.002 | 0.38 (0.19-0.76) | 0.006 | 0.37 (0.18-0.74) | 0.005 | 0.37 (0.19-0.75) | 0.006 |
| Smoking |  |  |  |  |  |  |  |  |  |  |  |
| 0 point | 12 | 141 | 8.5% | Referent |  | Referent |  | Referent |  | Referent |  |
| 1 point | 115 | 3,139 | 3.7% | 0.41 (0.22-0.76) | 0.005 | 0.44 (0.23-0.84) | 0.013 | 0.42 (0.22-0.82) | 0.010 | 0.41 (0.21-0.80) | 0.009 |
| Physical activity |  |  |  |  |  |  |  |  |  |  |  |
| 0 point | 36 | 867 | 4.2% | Referent |  | Referent |  | Referent |  | Referent |  |
| 1 point | 91 | 2,413 | 3.8% | 0.91 (0.61-1.34) | 0.618 | 1.09 (0.72-1.64) | 0.695 | 1.16 (0.76-1.78) | 0.495 | 1.13 (0.74-1.74) | 0.572 |
| Sleep |  |  |  |  |  |  |  |  |  |  |  |
| 0 point | 94 | 1,629 | 5.8% | Referent |  | Referent |  | Referent |  | Referent |  |
| 1 point | 33 | 1,651 | 2.0% | 0.33 (0.22-0.50) | <0.001 | 0.41 (0.27-0.63) | <0.001 | 0.42 (0.27-0.65) | <0.001 | 0.42 (0.27-0.65) | <0.001 |
| Stress |  |  |  |  |  |  |  |  |  |  |  |
| 0 point | 84 | 1,679 | 5.0% | Referent |  | Referent |  | Referent |  | Referent |  |
| 1 point | 43 | 1,601 | 2.7% | 0.52 (0.36-0.76) | 0.001 | 0.77 (0.52-1.14) | 0.197 | 0.76 (0.51-1.13) | 0.172 | 0.76 (0.51-1.14) | 0.185 |
| Fatigue |  |  |  |  |  |  |  |  |  |  |  |
| 0 point | 91 | 1,682 | 5.4% | Referent |  | Referent |  | Referent |  | Referent |  |
| 1 point | 36 | 1,598 | 2.3% | 0.40 (0.27-0.60) | <0.001 | 0.62 (0.40-0.95) | 0.027 | 0.63 (0.41-0.97) | 0.035 | 0.63 (0.41-0.97) | 0.034 |
| Diet |  |  |  |  |  |  |  |  |  |  |  |
| 0 point | 76 | 1,549 | 4.9% | Referent |  | Referent |  | Referent |  | Referent |  |
| 1 point | 51 | 1,731 | 2.9% | 0.59 (0.41-0.85) | 0.004 | 0.67 (0.46-0.98) | 0.039 | 0.67 (0.45-0.97) | 0.036 | 0.66 (0.45-0.97) | 0.036 |
| Body mass index |  |  |  |  |  |  |  |  |  |  |  |
| 0 point | 58 | 1,669 | 3.5% | Referent |  | Referent |  | Referent |  | Referent |  |
| 1 point | 69 | 1,611 | 4.3% | 1.24 (0.87-1.77) | 0.231 | 1.24 (0.87-1.79) | 0.238 | 1.22 (0.85-1.76) | 0.284 | 1.24 (0.85-1.79) | 0.263 |
| Alcohol |  |  |  |  |  |  |  |  |  |  |  |
| 0 point | 77 | 1,956 | 3.9% | Referent |  | Referent |  | Referent |  | Referent |  |
| 1 point | 50 | 1,324 | 3.8% | 0.96 (0.67-1.38) | 0.816 | 1.16 (0.79-1.69) | 0.446 | 1.16 (0.80-1.70) | 0.439 | 1.16 (0.80-1.70) | 0.432 |
| CLI (no smoking) |  |  |  |  |  |  |  |  |  |  |  |
| 0-2 points | 55 | 780 | 7.1% | Referent |  | Referent |  | Referent |  | Referent |  |
| 3 points | 26 | 724 | 3.6% | 0.49 (0.30-0.79) | 0.004 | 0.51 (0.32-0.83) | 0.006 | 0.51 (0.32-0.83) | 0.007 | 0.51 (0.32-0.83) | 0.007 |
| 4 points | 26 | 763 | 3.4% | 0.47 (0.29-0.75) | 0.002 | 0.49 (0.30-0.79) | 0.004 | 0.49 (0.30-0.80) | 0.004 | 0.50 (0.31-0.81) | 0.005 |
| 5 points | 10 | 605 | 1.7% | 0.22 (0.11-0.44) | <0.001 | 0.24 (0.12-0.47) | <0.001 | 0.24 (0.12-0.48) | <0.001 | 0.24 (0.12-0.47) | <0.001 |
| 6-7 points | 10 | 408 | 2.5% | 0.33 (0.17-0.66) | 0.002 | 0.36 (0.18-0.73) | 0.004 | 0.36 (0.18-0.71) | 0.004 | 0.36 (0.18-0.73) | 0.004 |
| CLI (no physical activity) |  |  |  |  |  |  |  |  |  |  |  |
| 0-2 points | 41 | 604 | 6.8% | Referent |  | Referent |  | Referent |  | Referent |  |
| 3 points | 36 | 697 | 5.2% | 0.75 (0.47-1.19) | 0.217 | 0.77 (0.48-1.22) | 0.259 | 0.75 (0.47-1.20) | 0.231 | 0.76 (0.47-1.20) | 0.235 |
| 4 points | 27 | 808 | 3.3% | 0.48 (0.29-0.78) | 0.003 | 0.50 (0.30-0.82) | 0.006 | 0.50 (0.30-0.82) | 0.006 | 0.50 (0.30-0.83) | 0.007 |
| 5 points | 13 | 694 | 1.9% | 0.26 (0.14-0.49) | <0.001 | 0.28 (0.15-0.52) | <0.001 | 0.28 (0.15-0.52) | <0.001 | 0.28 (0.15-0.53) | <0.001 |
| 6-7 points | 10 | 477 | 2.1% | 0.29 (0.15-0.59) | 0.001 | 0.32 (0.16-0.64) | 0.001 | 0.31 (0.15-0.62) | 0.001 | 0.31 (0.15-0.63) | 0.001 |
| CLI (no sleep) |  |  |  |  |  |  |  |  |  |  |  |
| 0-2 points | 26 | 412 | 6.3% | Referent |  | Referent |  | Referent |  | Referent |  |
| 3 points | 32 | 643 | 5.0% | 0.78 (0.46-1.32) | 0.355 | 0.81 (0.48-1.39) | 0.454 | 0.83 (0.49-1.43) | 0.504 | 0.83 (0.48-1.43) | 0.503 |
| 4 points | 34 | 902 | 3.8% | 0.58 (0.34-0.98) | 0.043 | 0.63 (0.37-1.07) | 0.085 | 0.64 (0.37-1.09) | 0.098 | 0.63 (0.37-1.08) | 0.095 |
| 5 points | 22 | 775 | 2.8% | 0.43 (0.24-0.78) | 0.005 | 0.48 (0.27-0.87) | 0.015 | 0.48 (0.27-0.87) | 0.016 | 0.48 (0.26-0.88) | 0.017 |
| 6-7 points | 13 | 548 | 2.4% | 0.36 (0.18-0.71) | 0.003 | 0.41 (0.21-0.83) | 0.013 | 0.41 (0.20-0.83) | 0.013 | 0.41 (0.20-0.83) | 0.013 |
| CLI (no stress) |  |  |  |  |  |  |  |  |  |  |  |
| 0-2 points | 26 | 386 | 6.7% | Referent |  | Referent |  | Referent |  | Referent |  |
| 3 points | 40 | 682 | 5.9% | 0.86 (0.52-1.44) | 0.571 | 0.89 (0.53-1.48) | 0.654 | 0.89 (0.53-1.48) | 0.644 | 0.89 (0.53-1.49) | 0.655 |
| 4 points | 28 | 859 | 3.3% | 0.47 (0.27-0.81) | 0.006 | 0.49 (0.28-0.86) | 0.012 | 0.49 (0.28-0.86) | 0.013 | 0.49 (0.28-0.86) | 0.013 |
| 5 points | 21 | 807 | 2.6% | 0.37 (0.21-0.67) | 0.001 | 0.40 (0.22-0.73) | 0.003 | 0.41 (0.22-0.74) | 0.003 | 0.41 (0.22-0.74) | 0.003 |
| 6-7 points | 12 | 546 | 2.2% | 0.31 (0.15-0.62) | 0.001 | 0.34 (0.17-0.70) | 0.003 | 0.34 (0.17-0.69) | 0.003 | 0.34 (0.17-0.70) | 0.003 |
| CLI (no fatigue) |  |  |  |  |  |  |  |  |  |  |  |
| 0-2 points | 23 | 379 | 6.1% | Referent |  | Referent |  | Referent |  | Referent |  |
| 3 points | 39 | 660 | 5.9% | 0.97 (0.57-1.65) | 0.917 | 1.02 (0.60-1.73) | 0.954 | 1.04 (0.61-1.78) | 0.892 | 1.04 (0.61-1.79) | 0.876 |
| 4 points | 29 | 885 | 3.3% | 0.52 (0.30-0.92) | 0.024 | 0.57 (0.32-1.00) | 0.048 | 0.58 (0.33-1.02) | 0.059 | 0.58 (0.33-1.02) | 0.059 |
| 5 points | 24 | 830 | 2.9% | 0.46 (0.26-0.83) | 0.009 | 0.50 (0.28-0.91) | 0.023 | 0.51 (0.28-0.93) | 0.027 | 0.51 (0.28-0.94) | 0.029 |
| 6-7 points | 12 | 526 | 2.3% | 0.36 (0.18-0.74) | 0.005 | 0.41 (0.20-0.84) | 0.015 | 0.40 (0.20-0.83) | 0.014 | 0.41 (0.20-0.84) | 0.016 |
| CLI (no diet) |  |  |  |  |  |  |  |  |  |  |  |
| 0-2 points | 28 | 429 | 6.5% | Referent |  | Referent |  | Referent |  | Referent |  |
| 3 points | 42 | 721 | 5.8% | 0.89 (0.54-1.45) | 0.631 | 0.91 (0.56-1.50) | 0.714 | 0.92 (0.56-1.51) | 0.743 | 0.92 (0.56-1.51) | 0.736 |
| 4 points | 26 | 804 | 3.2% | 0.48 (0.28-0.83) | 0.008 | 0.51 (0.29-0.88) | 0.015 | 0.51 (0.30-0.89) | 0.018 | 0.51 (0.30-0.90) | 0.019 |
| 5 points | 17 | 741 | 2.3% | 0.34 (0.18-0.62) | 0.001 | 0.36 (0.19-0.66) | 0.001 | 0.37 (0.20-0.68) | 0.002 | 0.37 (0.20-0.69) | 0.002 |
| 6-7 points | 14 | 585 | 2.4% | 0.35 (0.18-0.68) | 0.002 | 0.38 (0.20-0.74) | 0.005 | 0.39 (0.20-0.75) | 0.005 | 0.39 (0.20-0.76) | 0.005 |
| CLI (no body mass index) |  |  |  |  |  |  |  |  |  |  |  |
| 0-2 points | 38 | 488 | 7.8% | Referent |  | Referent |  | Referent |  | Referent |  |
| 3 points | 33 | 616 | 5.4% | 0.67 (0.41-1.09) | 0.104 | 0.70 (0.43-1.13) | 0.142 | 0.72 (0.44-1.17) | 0.185 | 0.71 (0.44-1.16) | 0.170 |
| 4 points | 30 | 801 | 3.7% | 0.46 (0.28-0.75) | 0.002 | 0.49 (0.30-0.80) | 0.005 | 0.50 (0.30-0.82) | 0.006 | 0.50 (0.30-0.83) | 0.007 |
| 5 points | 12 | 726 | 1.7% | 0.20 (0.10-0.38) | <0.001 | 0.21 (0.11-0.41) | <0.001 | 0.22 (0.11-0.42) | <0.001 | 0.21 (0.11-0.42) | <0.001 |
| 6-7 points | 14 | 649 | 2.2% | 0.26 (0.14-0.49) | <0.001 | 0.29 (0.15-0.55) | <0.001 | 0.29 (0.15-0.55) | <0.001 | 0.29 (0.15-0.55) | <0.001 |
| CLI (no alcohol) |  |  |  |  |  |  |  |  |  |  |  |
| 0-2 points | 33 | 433 | 7.6% | Referent |  | Referent |  | Referent |  | Referent |  |
| 3 points | 34 | 602 | 5.6% | 0.73 (0.44-1.19) | 0.205 | 0.75 (0.46-1.23) | 0.251 | 0.74 (0.45-1.21) | 0.231 | 0.74 (0.45-1.22) | 0.232 |
| 4 points | 32 | 843 | 3.8% | 0.48 (0.29-0.79) | 0.004 | 0.50 (0.30-0.83) | 0.008 | 0.50 (0.30-0.84) | 0.008 | 0.50 (0.30-0.83) | 0.008 |
| 5 points | 13 | 717 | 1.8% | 0.22 (0.12-0.43) | <0.001 | 0.24 (0.12-0.46) | <0.001 | 0.24 (0.12-0.46) | <0.001 | 0.24 (0.12-0.46) | <0.001 |
| 6-7 points | 15 | 685 | 2.2% | 0.27 (0.15-0.51) | <0.001 | 0.30 (0.16-0.56) | <0.001 | 0.29 (0.15-0.55) | <0.001 | 0.30 (0.16-0.56) | <0.001 |
| CLI-weak components ^d^ |  |  |  |  |  |  |  |  |  |  |  |
| 0-1 points | 41 | 860 | 4.8% | Referent |  | Referent |  | Referent |  | Referent |  |
| 2 points | 43 | 1,260 | 3.4% | 0.71 (0.46-1.09) | 0.118 | 0.75 (0.48-1.16) | 0.190 | 0.76 (0.49-1.18) | 0.226 | 0.76 (0.49-1.19) | 0.233 |
| 3-4 points | 43 | 1,160 | 3.7% | 0.77 (0.50-1.19) | 0.239 | 0.85 (0.55-1.33) | 0.482 | 0.86 (0.55-1.34) | 0.497 | 0.86 (0.55-1.34) | 0.500 |

OR, odds ratio; CI, confidence interval; CLI, combined lifestyle index.

^a^: Model I adjusted for age. In the analyses of each individual component, the other components were further adjusted.

^b^: Model II adjusted for age, marital status, living condition, educational level, family income, employment status, menstrual age, and menopause. In the analyses of each individual component, the other components were further adjusted.

^c^: Model III adjusted for variables in Model II, hypertension, diabetes, hyperlipidaemia, myocardial infarction, stroke, and cancer. In the analyses of each individual component, the other components were further adjusted.

^d^: Four lifestyle factors (physical activity, stress, body mass index, and alcohol) that were not independently associated with migraine were included in the CLI-weak components.
